# Preference‒performance linkage in the diamondback moth, *Plutella xylostella* , and implications for its management

**DOI:** 10.1093/jis/14.1.85

**Published:** 2014-07-07

**Authors:** Marchioro Marchioro, Luís Amilton Foerster

**Affiliations:** Department of Zoology, Universidade Federal do Paraná, Curitiba, Paraná, Brazil

**Keywords:** host selection, enemy-free space, intrinsic rate of increase, oviposition behavior

## Abstract

Host plants affect development, survival, and reproduction of phytophagous insects. In the case of holometabolous species, whose larvae have little mobility to find a host plant, the ability of females to discriminate hosts on the basis of their nutritional quality may be an important factor determining insect performance. The preference‒performance correlation hypothesis states that females will choose to lay their eggs on host plants that provide the best offspring performance. The effects of three cultivated and two wild brassicas (Brassicales: Brassicaceae) on the biology of the diamondback moth, *Plutella xylostella* L. (Lepidoptera: Plutellidae), an important pest of brassicas, were investigated. Based on these data, the preference–performance correlation hypothesis was tested. The results allowed the discussion of the possible role of wild brassicas on population dynamics of the pest. The life table parameters net reproduction rate and intrinsic rate of increase were used as indicatives of insect performance because they provide a detailed description of the survivorship, development, and reproduction of a population. Development, survival, and reproduction were affected by the cultivated and wild brassicas. Both net reproduction rate and intrinsic rate of increase were lower in individuals fed on wild brassicas, which indicates that brassicas are not nutritionally suitable for *P. xylostella* . Nevertheless, females showed no oviposition preference among host plants. The results showed that host plant quality might not be the only factor determining host selection by female *P. xylostella* . Results also suggest that wild brassicas may serve as a refuge for *P. xylostella* , favoring pest survival when crops are disturbed by insecticide application, irrigation, or ploughing.

## Introduction

The selection of host plants is one of the central debates in the study of the ecology and evolution of insect-plant interactions. Questions such as the origins of host shifts, the potential for sympatric speciation, the pattern of attack on host plants within local populations, and the causes of host specificity and offspring success have been widely discussed in the context of oviposition behavior ( Thompson and Pellmyr 1991 ).

Even in species with no parental care, when and where females place their offspring is one of the most important determinants of offspring success ( Bernardo 1996 ). An herbivorous female that lays its eggs on an appropriate plant is likely to produce offspring with higher fitness than a female that oviposits randomly ( Mousseau and Fox 1998 ). This is particularly important for holometabolous insects, which have larvae with relatively limited mobility to select a host plant; and in this case, the female choice can be considered the initial determinant of offspring fitness ( Renwick and Chew 1994 ). Because plant quality influences larval performance and adult reproductive potential ( Awmack and Leather 2002 ), host suitability is an important factor influencing host selection in many species.

The ability of females to select host plants that will provide the best offspring performance has been investigated, and the evidence for this correlation ranges from excellent to poor (Graton and Welter 1998). Most of the studies on the preference-performance correlation of insects have focused on the effect of the host plant on larval performance ( Roininen and Tahvanainen 1989 , Gratton and Welter 1998 , Martin et al. 2005 , Gillespie and Wratten 2011 ), and little attention has been given to reproduction. In this case, performance can only be measured at an individual level. By assessing the influence of host plants on reproductive parameters, however, population level measures of performance can be calculated by using life table parameters. Although life table parameters are useful to predict population growth potential, this is rarely done when evaluating host suitability in the context of the preference-performance hypothesis.

The diamondback moth, *Plutella xylostella* L. (Lepidoptera: Plutellidae), is the most important pest attacking brassica (Brassicales: Brassicaceae) crops worldwide ( Talekar and Shelton 1993 ). In Brazil, *P. xylostella* is a serious pest in the major brassica-producing areas of the country. The diversity and abundance of host plants are among the reasons cited for the high population levels of *P. xylostella.* The diamondback moth feeds on wild and cultivated brassicas, but only in the absence of cultivated hosts does the pest appear to maintain itself on wild species ( Talekar and Shelton 1993 ). In temperate regions, wild brassicas are used as hosts by *P. xylostella* when suitable crops are absent ( Harcourt 1986 , Talekar and Shelton 1993 ). In subtropical and tropical regions, brassicas are grown throughout the year, and therefore the role of wild species in pest population dynamics is probably different from that found in temperate regions.

Studies on the effects of cultivated and wild brassicas on the performance of *P. xylostella,* and its relationship with oviposition choice, may help to clarify the role of wild brassicas in pest population dynamics. Studying the development and reproduction of *P. xylostella* on these hosts is important in determining whether the insect survives and reproduces on wild brassicas. This also can help to understand which life history parameters are influenced by larval diet and their impact on pest performance. Ultimately, an investigation of *P. xylostella* preference-performance correlation can shed light on whether females lay eggs on wild hosts even when cultivated brassicas are available. Thus, this study aimed to evaluate the relationship between host quality and oviposition preference of *P. xylostella.* Because it is known that the nutritional quality of a diet affects insect fitness, it is expected that females of *P. xylostella* choose to lay their eggs on host plants that provide the best offspring performance.

## Materials and Methods

### Laboratory rearing of *P. xylostella*

A colony of *P. xylostella* was established from larvae and pupae collected in organic commercial crops of broccoli *(Brassica oleracea* L. cv. *italica)* and cauliflower *(B. oleracea* L. cv. *botrytis)* in Colombo, Paraná State, southern Brazil (25° 17' S, 49° 13' W). Periodically, immatures and adults were collected and added to the laboratory colony to maintain the genetic diversity of the stock. The insects were maintained in climatic chambers (model 347 CDG; FANEM, www.fanem.com .br) regulated at 20 ± 1°C, 70% ± 10% RH, and a photoperiod of 12:12 L:D. The rearing methods were the same as those described by Marchioro and Foerster (2011) .

### Host plants

The biology and oviposition preferences of *P. xylostella* were evaluated on three cultivated brassicas, cabbage *(B. oleracea* var. *capitata* L., cv. Fuyutoyo, Sakata Sementes), broccoli *(B. oleracea* var. *italica* L. cv BRO68, Syngenta) and cauliflower *(B. oleracea* var. *botrytis* L. cv. *Barcelona,* Horticeres), and two wild species, wild radish *(Raphanus raphanistrum* L.) and turnipweed *(Rapistrum rugosum* (L.)). The wild brassicas were selected on the basis of their common occurrence near commercial crops in southeastern Brazil.

### Larval performance

For each host plant, 60 newly hatched larvae were individually separated with a fine bristle brush into polyethylene vials (2 cm diameter and 4 cm height) containing fresh leaves of the host plant. The fresh leaves of the same age offered to the larvae were cut into pieces of equal sizes ( **∽** 9 cm ^2^ ). Every two days, food was changed and the vials were cleaned to remove frass. The insect development stage and mortality were checked daily. The parameters used to evaluate larval performance were development time and survival of the immature stages.

### Reproduction and fitness

Adults obtained from the experiments on larval performance were used to evaluate the influence of wild and cultivated brassicas on reproduction. Twenty replicates were tested for each host plant, and each replicate comprised one female/male pair kept in cylindrical plastic cages (10 cm diam, and 10 cm high), with the base lined with paper towel. Adults were fed on 10% honey solution diluted in distilled water provided in a cotton ball. Because females of *P. xylostella* lay their eggs only in the presence of a host, a piece of host plant leaf was used as a stimulant to induce oviposition behavior. This piece of host plant leaf (5x5 cm) was placed between two superposed lids, and the lower lid had an opening (4x4 cm) in the center to allow adults to make contact with the leaf inside the cage. The internal surface of the inner lid was lined with sulfite paper, where the eggs were laid, around the exposed host plant leaf. The eggs were counted daily and separated in plastic vials according to the female and oviposition date. The date of hatching and the number of hatched larvae were recorded to determine the incubation period and egg viability. The length of preoviposition and oviposition periods, longevity of males and females, lifetime fecundity, and egg viability were used as parameters to evaluate the effects of host plant on reproduction.

### Fertility life table parameters

Fertility life tables were created for each host plant to measure offspring performance. The reproductive parameters calculated were the net reproductive rate *(R0),* intrinsic rate of increase *(r _m_ ),* and the duration of a generation ( *T* ), according to Carey (1993) . The sex ratio used in the construction of the life tables was the one recorded for each host in the larval performance experiment: 0.55, 0.51, 0.57, 0.49, and 0.52 for cabbage, broccoli, cauliflower, wild radish, and turnipweed, respectively.

### Oviposition preference tests

The adults used in the oviposition preference tests derived from the colony. Adults were fed during larval stage on kale leaves *(B. oleracea* L. var *acephala),* a host not assessed in the oviposition preference tests.

Female oviposition preference was assessed by using two- and multiple-choice tests. In two-choice tests, all possible paired combinations between wild and cultivated brassicas were tested. The tests were performed in cylindrical plastic cages (10 cm diameter and 20 cm high) containing one excised leaf of each host in the edges of the cage. The host plants were exposed for 48 hr to a newly emerged pair released in the center of the cage. Pretests showed that within a 48-hr period, the pairs had time to mate and lay eggs on their preferred host. After 48 hr, all eggs laid on each host plant were counted. Altogether, 20 replicates were used for each possible combination, with one cage representing a replicate of that combination.

In the multiple-choice test, adult *P. xylostella* were exposed simultaneously to three cultivated brassicas and one wild brassica. Excised leaves of the host plants were kept in plastic vials with water to keep the leaves hydrated. To prevent adults from falling into the water, the plastic vials were sealed with plastic film containing a hole where the plant petiole was inserted. Host plant leaves were arranged randomly and equally spaced within wooden cages (45 **x** 30 **x** 31 cm) with the top screened and exposed to four newly emerged pairs for 48 hr. Altogether, 10 replicates were used for each of the two possible combinations, and one cage was considered as one replicate.

During the preference tests, adults were fed on 10% honey solution provided in a cotton ball. All experiments in this study were performed in climatic chambers regulated at 20 ± 1ºC, 70 ± 10% RH, and a 12:12 L:D photoperiod.

### Statistical analysis

The effects of wild and cultivated brassicas on *P. xylostella* biology and oviposition preferences in the multiple-choice test were evaluated using analysis of variance (ANOVA). The normality of the data was tested using the Shapiro-Wilk’s W test before performing ANOVA. Oviposition preference in two-choice tests was compared using Student’s *t-* test *(P* < 0.05). Survival rate from egg to adult stage was compared using chi-square test *(P* < 0.05). In addition, the Kaplan-Meier analysis was performed to compare survival of males and females on the evaluated host plants ( Kaplan and Meier 1958 ) *(P* < 0.05).

A stepwise-forward discriminant analysis was used to screen out the variables that allow differentiation among hosts. The stepwise-forward discriminant analysis begins with no variables in the model, and at each step a variable is included based on its discriminatory significance determined by the Fischer test ( *F* ). The input parameters for the discriminant analysis were group variables (cabbage, broccoli, cauliflower, wild radish, and turnipweed) and the continuous independent variables (history life traits measured in the study). Only the individuals monitored from hatching to adult death were used in the discriminant analysis. All statistical tests were performed with the software Statistica 8 ( Statsoft 2008 ).

## Results

### Development time and survival rate

**Figure 1.**
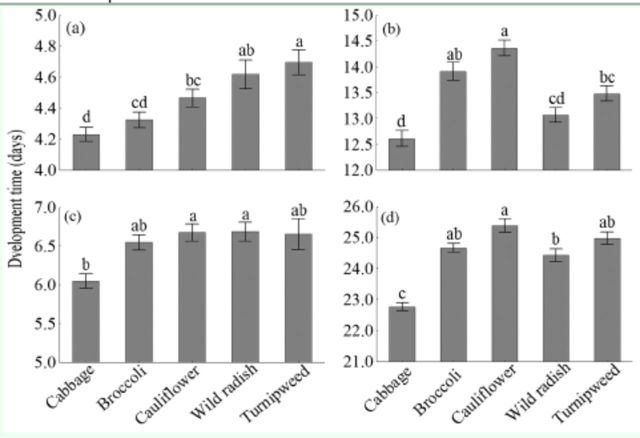
Mean development time (± SEM) in days of the stages of egg (a), larvae (b), pupae (c), and total immature stages (d) of *Plutella xylostella* . Means followed by the same letter are not significantly different from each other according to ANOVA, Tukey’s HSD test ( *P >* 0.05). High quality figures are available online.

Development times of eggs ( *F*_(4,89)_ = 13.29, *P* < 0.001), larvae ( *F*_(4,284)_ = 19.91, *P* < 0.001), pupae ( *F*_(4,264)_ = 2.76, *P* = 0.028), and egg‒adult cycle ( *F*_(4,264)_ = 36.88, *P* < 0.001) were significantly affected by the host plants ( Fig. 1a , b, c, d). The development time of the immature stages, as well as the time from egg to adult, was shortest when individuals fed on cabbage, and longest on cauliflower and turnipweed ( Fig1. 1a , b, c, d).

**Figure 2.**
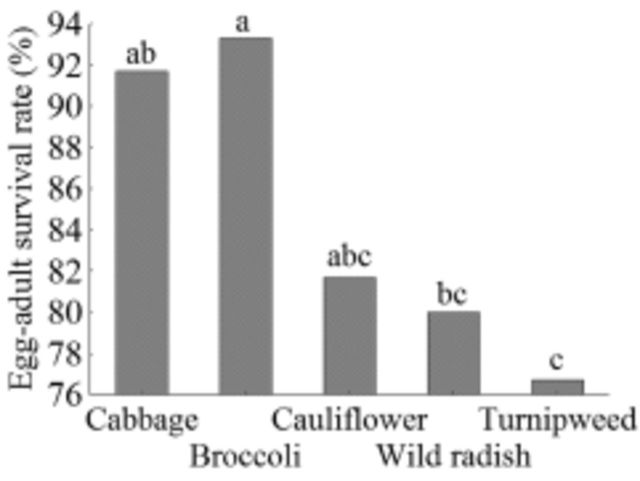
Percentage of *Plutella xylostella* that fed on different host plants reaching the adult stage. Values followed by the same letter are not significantly different from each other according to chi-square test ( *P* > 0.05).High quality figures are available online.

Host plants also affected the percentage of individuals reaching the adult stage. Survival rates on turnipweed (77%) and wild radish (80%) were significantly lower than on broccoli (93%; Fig. 2 ). Despite the significant differences between cultivated and wild brassicas, the percentage of individuals reaching the adult stage was >70% in all host plants. When the whole life cycle was considered in the Kaplan-Meier analysis, a significant effect of host plant was recorded only in the survival rate of females (χ² _4_ = 11.12, *P =* 0.025).

### Longevity and reproduction

**Figure 3.**
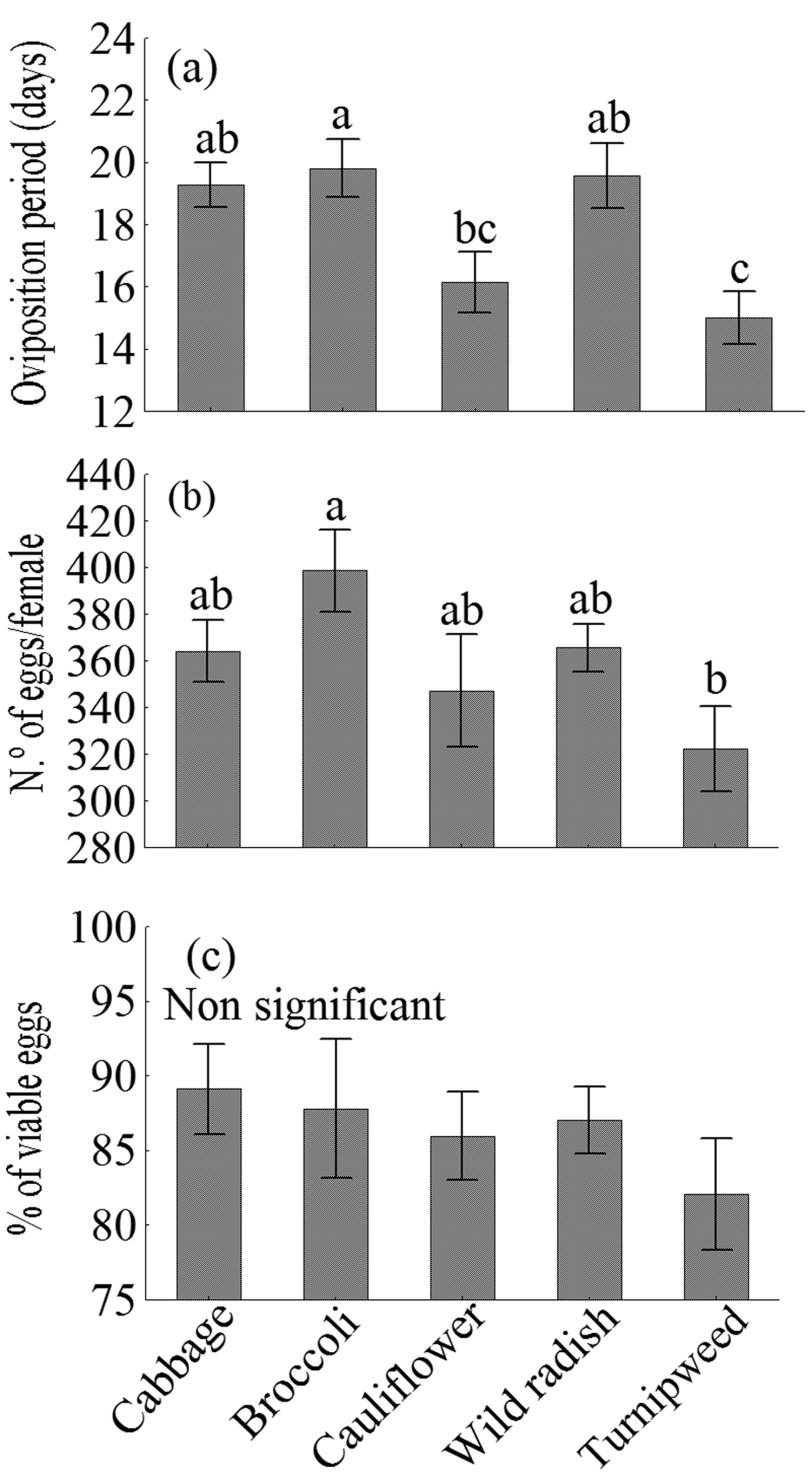
Oviposition period in days (a), lifetime fecundity (b), and percentage of viable eggs (c) (± SEM) of *Plutella xylostella* that fed on different host plants. Means followed by the same lower case letter are not significantly different from each other according to ANOVA, Tukey’s HSD test ( *P >* 0.05). High quality figures are available online.

Host plant significantly affected the length of the oviposition period *(F4,99 =* 6.02, *P* < 0.001) and lifetime fecundity *( F4,99 =* 2.65, *P* = 0.042) ( Fig. 3 ). When larvae fed on turnipweed, the oviposition period was significantly shorter than on cabbage, broccoli, and cauliflower.

**Figure 4.**
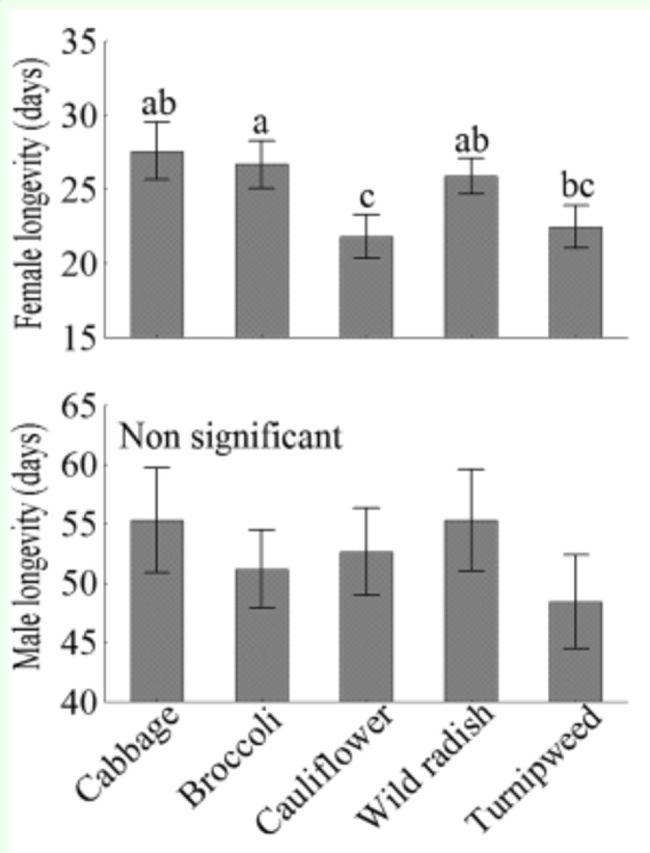
Mean longevity (± SEM) of females and males of *Plutella xylostella* that fed on different host plants. Means followed by he same lower case letter are not significantly different from each other according to ANOVA, Tukey’s HSD test ( *P >* .05). No significant differences between males and females were detected. High quality figures are available online.

The highest lifetime fecundity was recorded in individuals that fed on broccoli during the larval stage (399 eggs/female), which was significantly higher than those that fed on turnipweed (322 eggs/female). By contrast, the host plants did not affect egg viability *(F4,93* = 0.59, *P* = 0.673; Fig. 3 ). Longevity of females was affected by host plants *(F4 _,9_ 8 =* 2.80, *P* < 0.029), but no significant difference was recorded in the longevity of males *(F4,96 =* 0.75, *P* = 0.571). Males lived longer than females on all host plants *(F4 _,1_ 94 =* 225.80, *P* < 0.001; Fig. 4 ).

### Life table parameters

The net reproductive rate *(R0)* and the intrinsic rate of increase *(r _m_ )* were highest on cabbage and broccoli, whereas the generation time ( *T* ) was longest on the wild brassicas ( Table 1 ). The highest and lowest *R _0_* were provided by cabbage (141.4) and turnipweed (73.9), respectively. The *r _m_* was highest in broccoli (0.21) and lowest in wild radish and turnipweed (0.15).

**Table 1:**
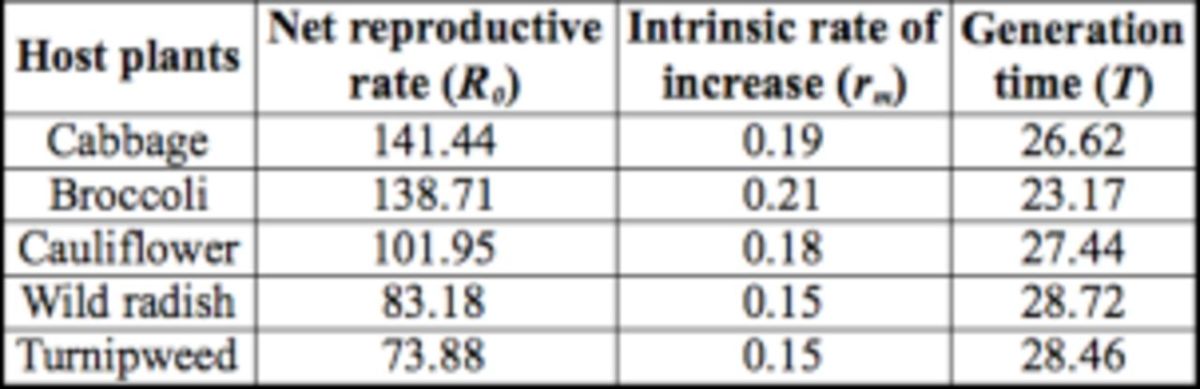
Estimated parameters of the life table of Plutella xylostella that fed on different host plants.

### Discriminant analysis

**Figure 5.**
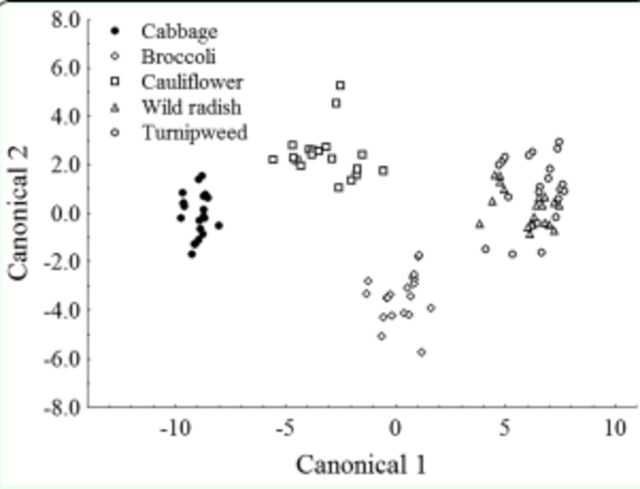
Graphical representation of the stepwise discriminant analysis: position of the cultivated and wild brassicas on the plane defined by the canonical variables 1 and 2. High quality figures are available online.

A significant model was obtained when developmental and reproductive parameters were used as explanatory variables to distinguish the five evaluated host plants (Wilk’s λ = 0.0045, *F _(24,311)_ =* 48.116, *P* < 0.001; Tables 2 and 3 ). Although the model did not clearly distinguish between individuals that fed on wild radish and turnipweed, the graphical representation of the host plants in the plane defined by the first two canonical functions demonstrates that cultivated brassicas can be separated from the wild species ( Fig. 5 ). The classification matrix of the model obtained indicates a correct global classification of 93.1%.

**Table 2:**
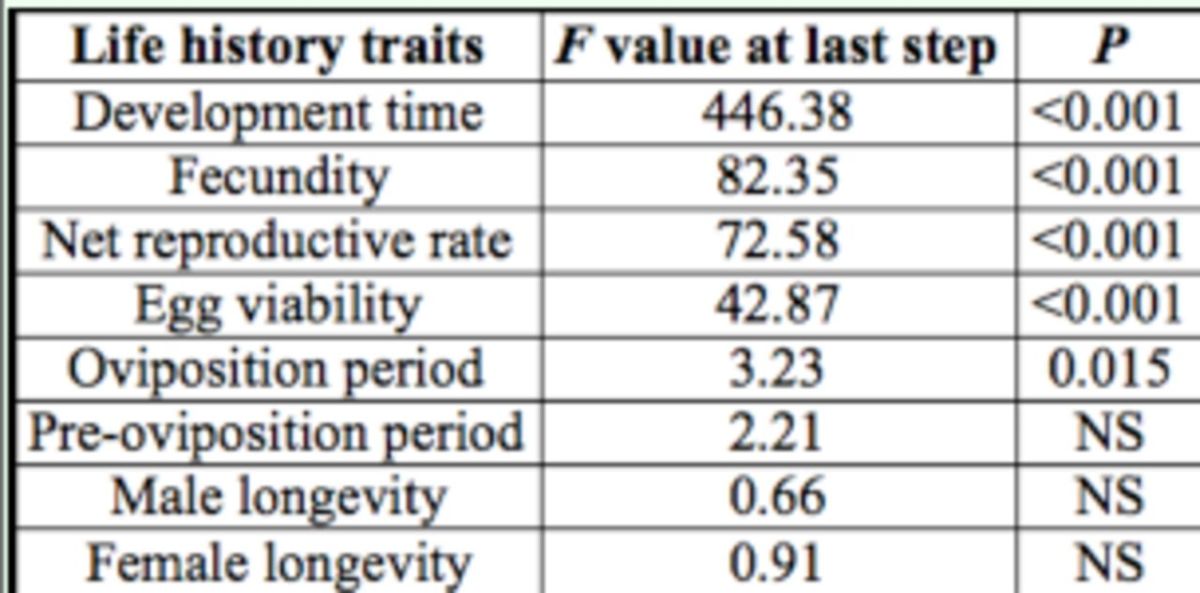
Power of discrimination of life history traits driving the discrimination of the five host plants at the end of the analysis.

**Table 3:**
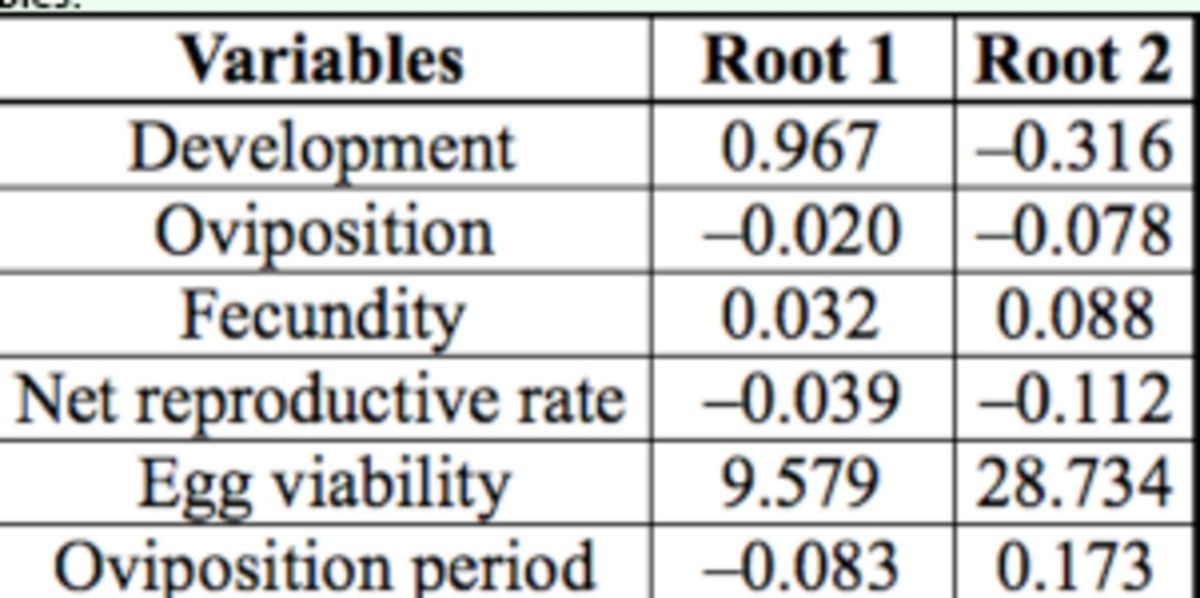
Coefficients for the most significant canonical variables.

### Oviposition preference

**Figure 6.**
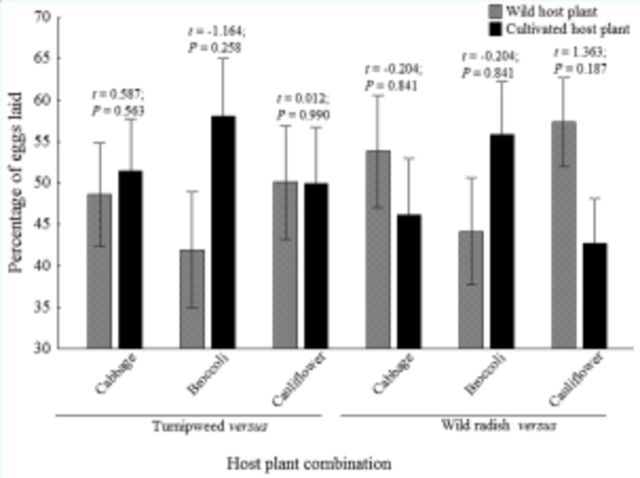
Mean percentage of eggs laid (± SEM) by *Plutella xylostella* on leaves of wild and cultivated brassicas in the two-choice test. High quality figures are available online.

**Figure 7.**
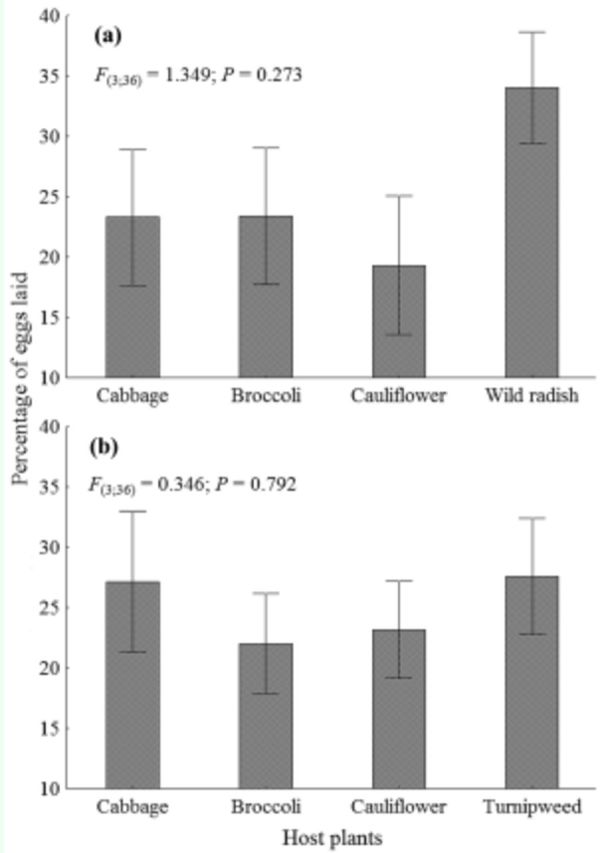
Mean percentage of eggs laid (± SEM) by *Plutella xylostella* on leaves of cultivated and wild brassicas in the multiple-choice test. (a) and (b) are respectively wild radish and turnipweed compared to cultivated brassicas. High quality figures are available online.

Females of *P. xylostella* showed no oviposition preference for the host plants evaluated in both two ( Fig. 6 ) and multiple-choice tests ( Fig. 7a , b). High individual variation in oviposition preference was indicated by the high standard error.

## Discussion

Development, survival, and reproduction of *P. xylostella* varied between wild and cultivated brassicas according to the discriminant analysis. An extension of development time and a reduction in survival rates and lifetime fecundity are typically considered an indication of a low-quality diet ( Waters and Barfield 1989 , Awmack and Leather 2002 ). Based on these parameters, wild radish and turnipweed were less suitable hosts for *P. xylostella* than cabbage and broccoli. Nevertheless, *P. xylostella* completed development from egg to adult on wild radish and turnipweed, suggesting that these wild brassicas are adequate alternative hosts. Previous studies also recorded an extension in development time and lower survival rate and fecundity of *P. xylostella* that fed on wild compared with cultivated brassicas ( Ayalew et al. 2006 , Kahuthia-Gathu et al. 2008 , Saeed et al. 2010 ), but the consequences of these results on host choice by females was not assessed.

In addition to the nutritional quality, the morphological traits of the host plant also can affect development and survival of *P. xylostella* ( Sarfraz et al. 2006 ). The type and amount of epicuticular waxes are particularly important for neonate larvae of *P. xylostella* . Eigenbrode et al. (1990) showed that neonate larvae spent more time moving and searching and less time biting on glossy phenotypes of cabbage than on normal wax bloom varieties. This difference in behavior results in poorer larval survival, as confirmed by further studies that showed a clear relationship between survival and the type and amount of leaf waxes ( Eigenbrode et al. 1991 ). The amount and composition of epicuticular wax vary according to brassica species ( Rutledge and Eigenbrode 2003 ), which might explain, at least in part, the differences in *P. xylostella* survival among host plants in this study.

Because the intrinsic rate of increase ( *r _m_* ) and net reproductive rate ( *R _0_* ) provide the most detailed description of survivorship, development, and reproduction of a population, they are commonly used to assess the influence of an external factor such as larval diet on population growth (Chi and Yang 2003). Individuals that fed on cultivated brassicas recorded the highest *r _m_* and *R _0_* , showing that these hosts are more suitable for *P. xylostella* . The comparison of *r _m_* indicates the significant effect of host plants on population growth of *P. xylostella* . The *r _m_* estimated for wild radish, turnipweed (both 0.15), and broccoli (0.21) suggests that during one host plant cycle (∽90 days), population growth could be 37 times higher in larvae that fed on broccoli.

Along with insect performance, oviposition preference is another important factor that determines the ability of phytophagous insects to use a particular group of plants ( Futuyama and Peterson 1985 ). *P. xylostella* uses chemical and/or physical stimuli to locate and select its host ( Sarfraz et al. 2006 ). The glucosinolates are a key group of plant chemicals in the Brassicaceae family and are used by *P. xylostella* for host location and as a stimulant that induces oviposition behavior ( Sarfraz et al. 2006 ). The presence of epicuticular wax and pubescence also are important traits affecting host selection by females of *P. xylostella* ( Sarfraz et al. 2006 ). It is expected that in the cultivated species, the amount of defensive and secondary plant compounds may be severely altered as a result of the cultivation process, even though no clear oviposition preference was recorded among host plants.

Although without statistical significance, some trends were observed in female preference, such as the higher number of eggs laid on wild radish compared to cabbage and cauliflower in the two-choice tests. Also, in the multiple-choice test, females laid more eggs on wild radish than on the cultivated plants. This issue might be worth exploring under laboratory and field conditions to evaluate the potential of this wild brassica as a trap crop.

The results obtained in this study differ from those reported by Kahuthia-Gathu et al. (2008) about the preference of *P. xylostella* for two cultivated and four wild brassicas in laboratory. These authors observed a significant oviposition preference for wild species.

In our study, we used excised leaves of the host plants in the preference tests; it is questionable whether this affected the ability of females to discriminate hosts and resulted in the observed lack of preference. Studies with *P. xylostella* and other lepidopteran species have shown that females are able to discriminate host plants using excised leaves and show a hierarchy of preference similar to the one obtained with the entire plant ( Greenberg et al. 2002 , Kahuthia-Gathu et al. 2008 ). On the other hand, female preference for some species of wild brassicas may be influenced by emission of volatiles when the host plant is attacked by herbivores or is otherwise injured ( Zhang et al. 2008 ).

The ability of adults to discriminate hosts on the basis of their nutritional quality may be important in determining offspring performance ( Thompson 1988 ). Therefore, a positive preference–performance correlation is hypothesized as a result of a strong selection pressure for oviposition in hosts that maximize survival and population growth ( Thompson and Pellmyr 1991 ). The existence of such a relationship has been demonstrated in some species ( Hamilton and Zalucki 1993 , Obermaier and Zwöfer 1999 , Greenberg et al. 2002 ), but it appears to be weak in others ( Roininen and Tahvanainen 1989 , Valladares and Lawton 1991 , Underwood 1994 , Gripenberg et al. 2007, Clark et al. 2011 ). Females of *P. xylostella* did not discriminate among the evaluated host plants despite their clear influence on insect performance. Unlike our findings, Zhang et al. (2012) conducted a me-taanalysis study with other wild brassicas and found a positive relationship between oviposition preference and pest performance. The discrepancy between both studies may be due to the different methodology used, the host plants evaluated, or the geographic variation in oviposition preference ( Forister 2004 , Gotthard et al. 2004 ).

A weak or nonexistent preference– performance correlation may arise from several evolutionary, ecological, and/or life history factors ( Thompson 1988 , Larsson and Ekbom 1995 ). Because it may take many generations for natural selection to filter out females that choose poor-quality hosts, the preference–performance correlation can be weak when females interact with a novel or relatively rare suitable plant ( Thompson 1988 ). In the absence of a co-evolutionary history, females may select a host plant based on insufficient information and ignore the presence of important nutrients or substances that negatively affect insect development (Kahu-thia-Gathu et al. 2008). This seems to be the case for the wild brassica, *Barbarea vulgaris* . A greenhouse study showed that this species does not support larval development of *P. xylostella* , but females prefer to lay eggs on *B. vulgaris* instead of cabbage ( Badenes-Perez et al. 2004 ). Because *P. xylostella* is native to Africa ( Kfir 1998 ) and *B. vulgaris* is endemic to North America, it is possible that this interaction is recent.

Furthermore, neither female choice nor offspring performance happen in isolation; they take place in a more complex ecological context with an unequal distribution of host plants, competitors, and natural enemies ( Gripenberg et al. 2008 ). The distribution of other species may have significant effects on insect performance and females’ oviposition preference ( Gripenberg et al. 2008 , Van Mele et al. 2009 ). Although a plant may only be partially suitable as food for an insect, it can provide a relatively safe environment from natural enemies and competitors ( Atsatt 1981 , Ohsaki and Sato 1994 ).

In this study, the role of nutritional quality of the host as a determinant of offspring performance was highlighted; any top-down ecological factors that may exert an important selective pressure were not evaluated. It is known that the pressure of natural enemies on herbivores may vary among host plants ( Thompson 1988 ). This is true for larval parasitoids of *P. xylostella* that use plant signals to locate their host ( Potting et al. 1999 , Ohara et al. 2003 ), with different rates of parasitism observed, according to the plant on which the host insect feeds ( Liu et al. 2000 , Hasseeb et al. 2001). It is possible that the threat of predation and parasitism on populations of *P. xylostella* is a key factor influencing its performance and, as a consequence, the oviposition preference of females. In this case, host nutritional quality and top-down effects may act simultaneously on *P. xylostella* populations.

The results obtained in this study help in understanding the role of wild brassicas in the population dynamics of *P. xylostella* in southern Brazil. Females of this species laid their eggs indiscriminately, and populations developed on wild radish and turnipweed, suggesting that these species act as reservoirs for pest infestations when more preferable hosts are not available. If this occurs commonly in the field, wild brassicas may provide a refuge against disturbances that are known to cause mortality of *P. xylostella* in crop systems (e.g., insecticide spraying, irrigation, ploughing). Refuge areas are often used in pest management to provide microhabitats and resources for natural enemies ( Holland et al. 2000 ). Larval parasitoids of *P. xylostella* visit and feed on nectar of brassicaceous flowers, which increases their longevity and fecundity ( Gourdine et al *.* 2003 , Schellhorn et al. 2008 ). In this context, the development of control strategies based on habitat management should consider the benefits and risks associated with the presence of wild brassicas beside crops. The challenge is to use wild plants that are beneficial to natural enemies but are not used by *P. xylostella* as a host.

In conclusion, the study shows that wild and cultivated brassicas have differing effects on the performance of *P. xylostella* . Nevertheless, females showed no oviposition preference, suggesting that host suitability may not be the only factor determining host selection by *P. xylostella* . These results indicate that wild brassicas may sustain populations of the diamondback moth when cultivated species are not available or the crop is disturbed. Further studies should investigate the preference–performance correlation considering tritrophic interactions (plant-pest-natural enemy) to fully understand the role of bottom-up and top-down effects on host selection by *P. xylostella* .
